# Retinal vessel diameters and reactivity in diabetes mellitus and/or cardiovascular disease

**DOI:** 10.1186/s12933-017-0534-6

**Published:** 2017-04-26

**Authors:** R. Heitmar, G. Y. H. Lip, R. E. Ryder, A. D. Blann

**Affiliations:** 1 School of Life and Health Sciences, Aston University, Aston Triangle, Birmingham, B4 7ET UK; 2 Institute for Cardiovascular Sciences, University of Birmingham, City Hospital, Birmingham, B18 7QH UK; 3 Department of Diabetes and Endocrinology, City Hospital, Birmingham, B18 7QH UK

**Keywords:** Diabetes mellitus, Cardiovascular disease, Retinal vessel diameter, Retinal vessel reactivity, Blood glucose

## Abstract

**Background:**

Retinal vessel calibre and vascular dilation/constriction in response to flicker light provocation may provide a measure distinguishing patients suffering from diabetes mellitus and/or cardiovascular disease.

**Methods:**

One hundred and sixteen age and sex matched patients with diabetes mellitus (DM), cardiovascular disease (CVD) and both DM and CVD (DM + CVD) underwent systemic and intraocular pressure measurements. Retinal vessel calibres were assessed using a validated computer-based program to compute central retinal artery and vein equivalents (CRVE) from monochromatic retinal images. Vessel dilation and constriction responses to flicker light provocation were assessed by continuous retinal vessel diameter recordings. Plasma endothelial markers von Willebrand factor (vWf) and soluble E selectin (sEsel) were measured by ELISA.

**Results:**

Retinal vessel calibres were comparable across groups but CRVE correlated significantly with disease duration in DM patients (r = 0.57, p < 0.001). Patients suffering DM only exhibited reduced arterial vasomotion at rest and reduced arterial constriction following flicker light induced vessel dilation compared to patients with CVD and those suffering both CVD + DM (p = 0.030). Patients suffering from CVD + DM exhibited significant differences between each flicker cycle in regards to arterial maximum constriction (p = 0.006) and time needed to reach arterial maximum dilation (p = 0.004), whereas the other two groups did not show such inconsistencies between individual flicker cycles. vWf was raised in CVD + DM compared to the other two groups (p ≤ 0.02), whilst sEsel was raised in CVD + DM compared to DM alone (p = 0.044).

**Conclusions:**

Dynamic retinal vascular calibres as obtained by continuous diameter measurements using flicker light provocation can reveal subtle differences between groups suffering from CVD with and without DM. This difference in reaction pattern and lack of arterial constriction in DM may provide a suitable marker to monitor progression.

## Background

Cardiovascular disease (CVD) has a complex pathogenesis and can manifest as coronary artery disease (such as previous myocardial infarction and coronary artery stenosis/occlusion) cerebrovascular disease (leading to stroke) and peripheral artery disease (often requiring amputation). Diabetes mellitus (DM) is a major risk factor for CVD, but many diabetics also have other risk factors such as hypertension, and suffer from microvascular disease, such as of the retinal vessels, causing retinopathy [1, 2]. Leading clinical aspects of retinal vascular disease are disease duration and the degree of hyperglycaemia (as assessed by glycated haemoglobin [HbA_1c_]), and accordingly the accurate assessment of retinal function is a highly-sought after clinical tool [3, 4].

The retinal vasculature is readily accessible and can be observed non-invasively by fundus photography, video recording and tomographic technology. It’s potential as screening tool for cerebrovascular [5] and cardiovascular disease [6, 7] has been supported through large population studies [8–11]. These studies demonstrated a relationship between retinal vessel calibres and cardiovascular risk [11, 12]. Individuals with good cardiovascular health are less likely to have signs of retinopathy such as dilated retinal venules and narrow retinal arterioles, both of which are associated with increased risk of stroke and coronary artery disease [13].

While retinal vessel calibres provide only static indices, dynamic measurements such as retinal vessel reactivity to flicker light provocation can provide further insight into the status of the retinal microcirculation. Several authors have demonstrated a link between measures of cardiovascular health and retinal vessel dynamics, such as a decrease in retinal vessel dilation in presence of decreased flow mediated dilation of the brachial artery [14], prolonged reaction times in retinal arterial responses to flicker light in patients suffering from coronary artery disease [15], and decreased vessel dilation to flicker light in patients with coronary artery disease, and which depends on the severity of the disease [16]. Previous research has shown reduced retinal vessel dilation to flicker light in DM patients compared to controls which further reduced with the severity of diabetic retinopathy [17–19]. This lack of reactive hyperaemia in DM patients has been attributed to abnormal glial regulation of the retinal vasculature [20] as well as endothelial cell dysfunction leading to reduced nitric oxide (NO) synthase and sensitivity [17, 21]. While most studies conducted in DM patients examined both arteries and venous responses, they have not evaluated the full response to flicker light which includes the vasomotion at rest, vasoconstriction post flicker and reaction time.

Whilst retinal vessel dynamics have the potential as tool for risk stratification and screening as evidenced by the progressively decreasing vessel dilation with increasing severity of diabetic retinopathy, the relative influence of how different pathological combinations and risk factors impact on retinal vessel parameters is unclear. We hypothesised that retinal vessel responses to flicker light would be more adverse in those patients with both DM and CVD compared to patients with either DM or CVD alone.

## Methods

The study was approved by the National Health Service Research Ethics Committee East Midlands-Leicester (Ref: 12/EM/0062) and the Aston University Ethics Committee and adhered to the Declaration of Helsinki. Patients were recruited from the cardiovascular rehabilitation unit, hypertensive and diabetic outpatient clinics at City Hospital (Birmingham, UK). All patients gave written informed consent. Three groups were defined prior to recruitment: patients suffering from diabetes mellitus (type 1 and type 2) but free from cardiovascular: patients suffering from cardiovascular disease but free from diabetes, and patients suffering from both diabetes and cardiovascular disease. Diabetes was defined by attendance at a diabetes clinic and HbA_1c_ > 50 mmol/mol (6.7%). Cardiovascular disease was defined as history of myocardial infarction, coronary artery stenosis/occlusion, coronary artery bypass grafting, stroke, iliac/femoral artery stenosis or bypass or lower limb/foot/toe amputation. Exclusion criteria included recent (<3 month) myocardial infarction, stroke, or surgery, cancer, autoimmune disease (such as rheumatoid arthritis) or ophthalmological disease such as age-related macular degeneration. Serum endothelial markers von Willebrand factor (vWf) and soluble E selectin (sEsel) were measured by commercial ELISA (Dako, Ely, UK and R & D Systems, Abingdon, UK). Clinical, laboratory, medication and demographic data were collected and are shown in Table 1.

**Table 1:** Clinical data, medication and demographics

	DM (n = 36)	CVD (n = 43)	DM and CVD (n = 37)
DM type: 1/2	2/34	–	1/36
Ethnicity: CA/SA/B	28/4/4	37/4/2	32/5/0
Age (years)	64 (10)	64 (11)	65 (9)
Gender (m/f)	24/12	30/13	32/5
SBP (mmHg)	130 (13)	125 (19)	125 (16)
DBP (mmHg)	75 (9)	75 (13)	67 (10)*
HR (beats per minute)	76 (14)	67 (11)*	72 (16)
IOP (mmHg)	15 (3)^+^	13 (2)	14 (2)
BMI (kg/m^2^)	31 (6)	27 (4)*	31 (6)
HbA1c (mmol/mol)	58 (15)	42 (3)*	61 (20)
DM duration (years)	11 (4.5–16)	–	10 (4–17)
Medications			
Calcium channel blocker	20, 56%	13, 30%	14, 38%
ACEI/ARB	23, 64%	31, 72%	30, 81%
Metformin	26, 72%	–	21, 57%
Sulphonylurea	7, 19%	–	7, 19%
DPP-4 inhibitor	8, 22%	–	6, 16%
Insulin	15, 42%	–	14, 38%
GLP-1 agonist	4, 11%	–	4, 11%
Piaglitazone	4, 11%		2, 5%
Lipid-lowering	26, 72%	41, 95%	36, 97%
Aspirin	11, 31%	35, 81%	30, 81%
Clopidogrel	1, 3%	7, 16%	7, 19%
Nitrate	–	11, 26%	11, 30%
Oral anticoagulant	6, 17%	5, 12%	5, 14%
Beta-blocker	10, 28%	19, 44%	22, 59%
Diuretic	18, 50%	9, 21%	18, 49%
Thyroxine	6, 17%	6, 14%	3, 8%
Cardiovascular disease			
Coronary artery disease	–	31, 72%	30, 81%
Peripheral artery disease	–	5, 12%	4, 11%
Cerebrovascular disease	–	8, 19%	3, 8%

Data presented as mean with standard deviation, or as number of subjects and percentage

*CA* caucasian, *SA* South Asian, *B* black, *M* male, *f* female, *SBP* systolic blood pressure, *DBP* diastolic blood pressure, *HR* heart rate, *IOP* intraocular pressure, *BMI* body mass index, *HbA1C* glycated haemoglobin, *DM* diabetes mellitus, *ACEI* angiotensin-converting-enzyme inhibitor, *ARB* angiotensin receptor blockers, *DPP*-4 dipeptidyl peptidase-4 (‘gliptins’), *GLP*-1 glucagon-like peptide-1 (exenatide, liraglutide)

* Different compared to the two other groups (p < 0.01)

^+^ Different to the CVD group (p < 0.01)

Patients were advised to abstain from caffeinated drinks for a minimum of 12 h prior to their ocular examination. All patients were seated in a temperature-controlled room (21 ± 2 °C) for a minimum of 15–20 min to achieve a stable blood pressure (systolic blood pressure [SBP] and diastolic blood pressure [DBP] were measured using a digital sphygmomanometer [UA767, PMS Instruments, UK]). Intraocular pressure (IOP) was measured using a rebound t tonometer (I-Care, Mainline Instruments Ltd., UK) after which pupils were dilated with 1% tropicamide (Chauvin Pharmaceuticals Ltd., Kingston-Upon-Thames, UK).

### Dynamic and static retinal vessel assessment

**Fig. 1:**
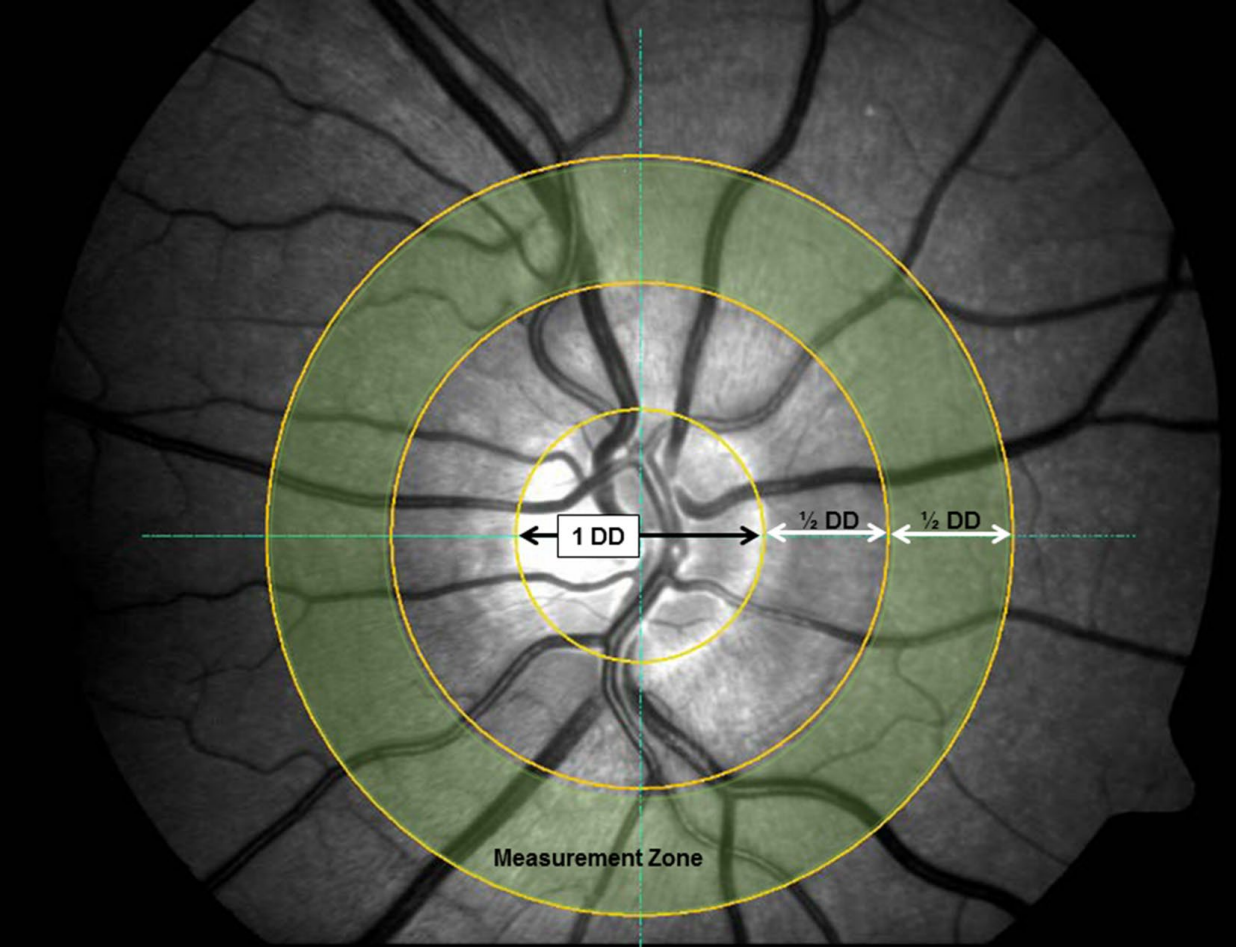
Sample image showing measurement area for static retinal vessel evaluation. *Central ring* denotes the diameter of the disc (1 DD), the *second* and *third circle* are each ½ DD separated to create a ½ DD wide* concentric ring* segment around the optic nerve head. This *ring segment* is highlighted here in *yellow* to illustrate the area in which retinal arterial and venous calibres are measured to calculate central retinal artery and vein equivalents (CRAE and CRVE)

Once pupils were fully dilated, digital fundus images and reactivity parameters of retinal arteries and veins to flicker light provocation were obtained from one eye of each patient [58 right eyes and 58 left eyes] (Retinal vessel analyser [RVA], Imedos Systems [UG] haftungsbeschraenkt Jena, Germany) [22]. For static vessel analysis monochromatic (red-free) fundus images were obtained, at a 30 °C camera angle with the optic nerve head (ONH) centred, using the inbuilt Zeiss FF450 + fundus camera (Zeiss GmbH, Germany), see Fig. 1. Summarised retinal vessel calibres of retinal arteries (central retinal artery equivalent, CRAE), veins (central retinal vein equivalent, CRVE) and the retinal artery-to-vein ratio (AVR) were calculated according to a standard protocol [23].

Dynamic imaging was followed by static imaging. Retinal vessel diameters were measured continuously at a sampling rate of 25 Hz. Stimulation of retinal blood vessels was done by optoelectronic interruption of the green fundus illumination used by the RVA resulting in a flicker light provocation with a 12.5 Hz frequency [24–26]. After BP stabilisation and image focussing one vessel segment of each, the superior temporal retinal artery and vein was chosen at a distance of 1.5–2 DD away from the margins of the ONH. Baseline diameters of both the artery and vein were recorded according to the standard RVA protocol [26] for 50 s and then followed by three cycles of 20 s flicker provocation with each 80 s recovery time. Resulting in a 350 s measuring period during which the fellow eye was occluded to improve patient fixation. From these diameter recordings of arteries and veins the values for baseline diameter fluctuation (BDF) maximum dilation (MD), maximum constriction (MC) and dilation amplitude (DA), time to reach MD following flickering light initiation (reaction time, RT) and arterial constriction time (CT = time to reach MC) were calculated for single flicker cycles and averaged responses [27].

**Fig. 2:**
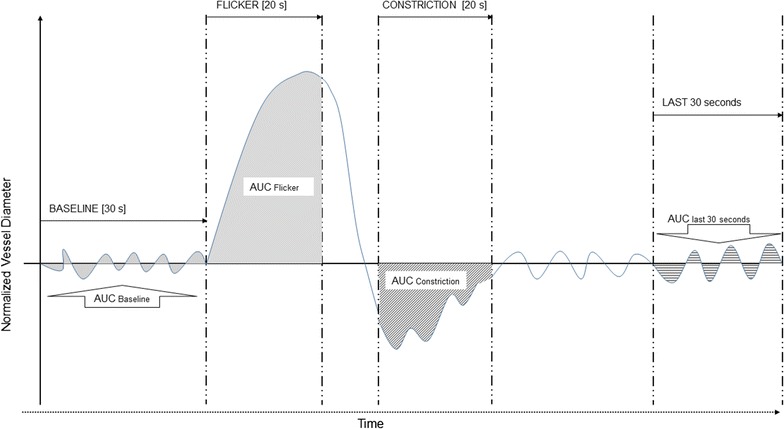
Schematic to illustrate area under the* curve* calculations. The *highlighted areas* in *grey* denote the time zones from which “Area under the* curve*” (AUC) data was calculated for baseline, during flicker, constriction and of the final 30 s

Area under the curve parameters of retinal arteriolar responses to FL were calculated using the averaged time course of all three FL cycles. AUC_Baseline_: area under the curve of the averaged 30 s prior to flicker start, AUC_20 s FL_: area under the curve of the averaged profile during 20 s flicker [28], AUC_20 s Constriction_: area under the curve of 20 s duration 10 s following flicker cessation and AUC_Last 30 s_: area under the curve of the last 30 s of the averaged reaction profile [29] (see Fig. 2).

### Statistical analysis

We tested the hypothesis that levels of a test statistic with a mean of 100 arbitrary units (standard deviation 14 units) would differ by 10% in patients with diabetes and cardiovascular disease compared with those patients with diabetes alone or cardiovascular disease alone. To achieve this at alpha <0.01 and 1-beta = 0.80 calls for data from 36 subjects per group. We therefore recruited consecutive patients until this sample size was achieved in each group. Associations between continuously-variable factors were sought using Spearman’s correlation. With this sample size per group, risks of types 1 and 2 statistical error are minimised when a correlation coefficient (r) exceeds 0.5.

Cross-sectional differences between the three groups were assessed using analysis of variance or the Kruskal–Wallis test, depending on distribution. Categorical data was analysed by the Chi squared test. Between group differences were sought using Tukey’s post hoc test. Prior to averaging retinal vessel responses of the three flicker cycles, within group analyses of the three flicker light cycles were sought using Friedman’s repeated (two-way) measures ANOVA. Comparisons between software generated dilatory parameters (the software generates three arterial parameters from averaged flicker responses [A_max_, A_min_ and A_peak_] and one venous response [V_max_]) and sequential diameter response analyses (SDRA) [27] were sought by paired *t* test whereas subgroup analyses of patients with diabetic retinopathy were compared using the Mann–Whitney-U test or t test (supplementary data). Statistical significance was set at p < 0.05, and results are reported as either mean (standard deviation) or as median (upper quartile-lower quartile). All data were analysed using Statistica Version 12.4 (Dell Software, Dublin, Ireland).

## Results

Table 1 shows clinical, demographic, medication and co-morbidity details of the 116 patients (36 DM only [2 Type 1 and 34 Type 2], 43 CVD only and 37 CVD and DM [1 Type 1 and 36 Type 2]). The three groups were matched for age (ANOVA p = 0.769) and sex (*X* ^2^ p = 0.110). Patients with DM and CVD had lower DBP than the other two groups, whilst heart rate, HbA1c and body mass index were lower in those with CVD alone. There was no difference in the distribution of the type of CVD between the two groups with this disease (*X* ^2^ p = 0.405). Table 2 shows retinal indices (summarized retinal vessel calibres and software generated vessel dynamic retinal vessel parameters). There were no significant differences between the three groups.

**Table 2:** Retinal parameters

	DM (n = 36)	CVD (n = 43)	DM and CVD (n = 37)	p value
CRAE (au)	178 (21)	175 (18)	175 (11)	0.660
CRVE (au)	213 (19)	208 (19)	215 (18)	0.212
AVR	0.84 (0.02)	0.85 (0.02)	0.83 (0.01)	0.389
A_max_ (%)	0.90 (0.45–2.45)	0.80 (−0.1 to 2.50)	0.90 (0.10–1.80)	0.558
A_min_ (%)	−0.40 (−0.85 to 0.05)	−0.50 (−1.10 to 0.20)	−0.50 (−1.10 to 0.10)	0.905
A_peak_ (%)	1.55 (0.40–3.25)	1.60 (0.30–2.90)	1.20 (0.20–2.40)	0.662
V_max_ (%)	3.25 (2.00–3.90)	3.10 (1.80–4.10)	2.50 (1.30–3.60)	0.206

Summarized retinal vessel calibres and software generated vessel reactivity parameters

Data presented as mean (SD) or median (lower quartile-upper quartile)

*Au* arbitrary units, *CRAE* central retinal artery equivalent, *CRVE* central retinal vein equivalent, *AVR* arterio-venous ratio, *A*_*max*_ IMEDOS software generated arterial maximum dilation to flicker, *A*_*min*_ IMEDOS software generated arterial maximum constriction following flicker, *A*_*peak*_ IMEDOS software generated arterial dilation amplitude, *V*_*max*_ IMEDOS software generated venous maximum dilation to flicker

p values by analysis of variance or the Kruskal–Wallis test as distribution demands

**Fig. 3:**
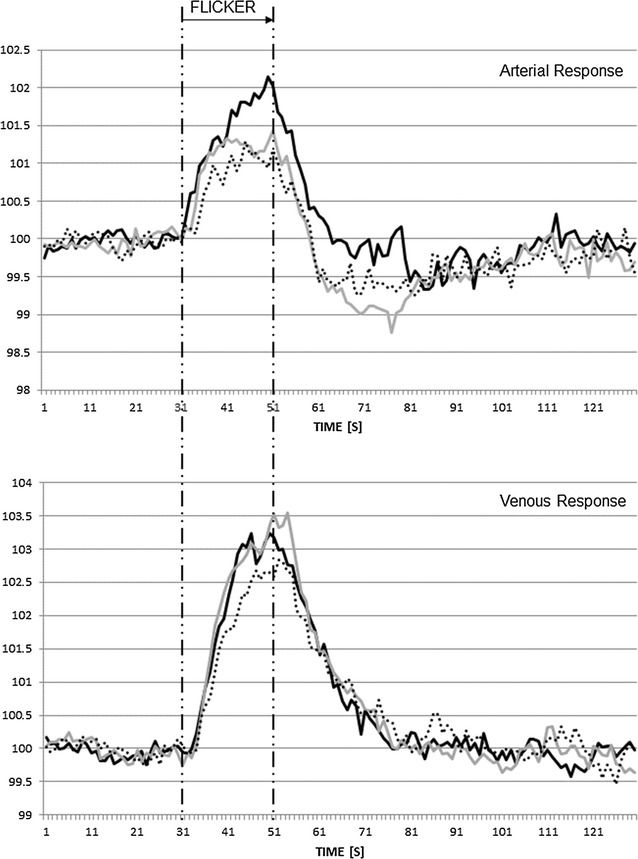
Averaged response to flicker light provocation of retinal vessels. Mean arterial (*top graph*) and retinal vein (*bottom graph*) responses for all three patient groups (*black solid line* DM only; *grey solid line* CVD; *black dotted line* DM and CVD)

Table 3 and Fig. 3 shows the averaged dynamic retinal vessel diameter and responses to flicker light stimulation of both arteries and veins. In cross-sectional analyses, the arterial BDF was lower in DM only group compared to the other two groups. The DM + CVD groups’ arterial maximum constriction following flicker cessation was inconsistent throughout the three flicker provocations, with the second cycles’ response being the largest compared to the first and third (−1.79; −2.50 and −1.68%; p = 0.006), this was not the case for the other two groups. The average arterial MC however was lower in those with DM alone compared to the two other groups. In single flicker analyses, the arterial RT was shortest in the second cycle than in the first and third cycles in the DM and CVD group only (20 s, 16 s, 23 s; p = 0.004). There were no such differences between individual flicker cycles in the other two groups.

**Table 3:** Dynamic arterial and venous vessel parameters

	DM (n = 36)	CVD (n = 43)	DM and CVD (n = 37)	p value
Arterial parameters				
A_SIZE_ (µm)	110 (16)	113 (20)	116 (18)	0.294
BDF (%)	1.66 (0.81)*	2.59 (1.95)	2.38 (1.38)	*0.019*
MD (%)	2.99 (2.23)	2.88 (2.29)	2.60 (1.68)	0.705
MC (%)	−1.45 (0.87)**	−2.06 (1.50)	−2.17 (1.52)	*0.030*
RT (s)	18 (11)	20 (12)	20 (12)	0.679
CT (s)	40 (10)	38 (8)	40 (7)	0.531
Venous parameters				
V_size_ (µm)	141 (20)	141 (19)	142 (18)	0.927
BDF (%)	1.91 (0.88)	2.53 (2.09)	2.45 (1.52)	0.192
MD (%)	4.34 (1.85)	4.28 (1.96)	4.16 (2.24)	0.928
MC (%)	−0.80 (1.20)	−0.79 (1.53)	−0.72 (1.15)	0.963
DA (%)	5.14 (2.25)	5.07 (2.22)	4.88 (2.64)	0.892
RT (s)	21 (9)	20 (4)	21 (7)	0.972

Retinal vessel reactivity parameters as calculated using sequential diameter response analysis (SDRA)

Data presented as mean (standard deviation) or median (inter quartile range)

All values given in percentage change to baseline vessel diameters or times in seconds

*BDF* baseline diameter fluctuation, *MD* maximum dilation, *MC* maximum constriction, *DA* dilation amplitude, *RT* reaction time, *CT* constriction time, *s* seconds

p values by analysis of variance (ANOVA)

* Different compared to CVD group (p < 0.05)

** Different compared to CVD and DM group (p < 0.05)

There was no difference between the three groups in regards of the venous responses to flicker light provocation. Table 4 shows area under the curve data: this index confirmed the significantly lower arterial vessel constriction in those with DM alone compared to the two other groups. The only significant correlation where the r value exceeded 0.45 was between the disease duration in patients with diabetes alone and the CRVE (r = 0.57, p < 0.001). Of the 73 patients with diabetes, six had diabetic retinopathy (according to UK DR grading^a^). With this small number, no analyses will be meaningful. Table 5 shows serum endothelial markers. vWf was higher in DM + CVD than in the other two groups, whilst sEsel was high in patients with DM + CVD versus patients with DM alone. In the DM alone group, vWf correlated with the venous reaction time in the first flicker cycle (r = 0.5, p = 0.003). In the DM + CVD group, sEsel correlated with CRVE (r = 0.54, p = 0.004) and with the AVR (r = −0.62, p = 0.001). There were no other statistically significant correlations.

**Table 4:** AUC data

	DM (n = 36)	CVD (n = 43)	DM and CVD (n = 37)	p value
AUC_Baseline data_	−0.9 (2.0)	−1.4 (2.2)	−1.0 (3.1)	0.704
AUC_20 s Flicker_	14.9 (6.0–30.1)	19.8 (−0.7–37.8)	12.0 (1.1–24.7)	0.429
AUC_20 s Constriction_	−4.0 (−15.7 to 3.8)*	−14.4 (−27.5 to −3.4)	−10.1 (−20.0 to −3.7)	*0.038*
AUC_Last 30 s_	−3.5 (18.9)	−6.7 (26.0)	−7.3 (23.1)	0.748

All data are presented as either means (± standard deviation) or median (upper quartile/lower quartile). Statistical significance is shown by bolded p-values. All values given in percentage change to baseline vessel diameters or times in seconds

*AUC*_*baseline*_ area under the curve of the averaged 30 s prior to flicker start, *AUC*_*20* *s FL*_ area under the curve of the averaged profile during 20 s flicker, *AUC*_*20s Constriction*_ area under the curve of 20 s duration 10 s following flicker cessation, *AUC*_*Last 30s*_ area under the curve of the last 30 s of the averaged reation profile

p value by analysis of variance or the Kruskal–Wallis test

* p < 0.05 lower than the two other groups

**Table 5:** Serum endothelial markers

	DM (n = 36)	CVD (n = 43)	DM and CVD (n = 37)	P value
Von Willebrand factor (IU/dL)	107 (21)^a^	111 (20)^b^	124 (22)	0.003
Soluble E selectin (ng/mL)	22 (8)^c^	26 (8)	27.5 (12)	0.03

Data mean (SD), p value from ANOVA

^a^ p = 0.004 to DM and CVD group

^b ^p = 0.02 to DM and CVD group

^c^ p = 0.044 to DM and CVD group (inter-group p values Tukey’s post hoc test)

## Discussion

We report several differences in retinal vessel physiology in those with DM alone, those with CVD alone, and those with both DM and CVD which confirm and extend those of others [16–18]. However, in our study, we found no differences in CRAE, CRVE, the AVR or IMEDOS indices in those with or without diabetes and/or cardiovascular disease. The reason for this negative finding could be that early changes due to inflammation as described by Virchow’s triad are present in all three groups. These alterations of the vascular system affecting blood flow, contributing to hypercoagulability and inducing vessel wall alterations through endothelial injury all contribute to an altered vessel structure over time but may present first by a change in vessel physiology prior to signs of structural damage.

Nevertheless, we found multiple differences to the responses of arteries to flicker stimulation, the most marked being those of the arterial MC and RT. These shared the property that responses with a difference in the second cycle compared to the first and third cycles in those with both DM and CVD, providing evidence of differences in the time course of arterial vessel dynamics in these pathologies. Averaged vessel diameter responses (as calculated by the software of the RVA and referred to as IMEDOS values) can mask differences not only in regards to reaction patterns but also underestimate the overall dilatory capacity [27]. While all three groups showed comparable results for summarised retinal vessel diameters and dynamic responses to flicker light patients suffering from DM alone exhibited increased vessel diameters (CRVE) with increased disease duration, confirming results of previous studies [30]. Furthermore, venous response to flicker light provocation was decreased with increasing disease duration. This decrease in venous dilation is in agreement with data described elsewhere [17, 18, 21, 30] and can be partly explained by the possibility that a pre-dilated vessel (as reflected by the increase in CRVE with increasing disease duration) has reduced residual dilatory capacity.

Area under the curve (AUC) analyses provides data that takes into account the time course as well as the extent of the vessel dilation recorded. The results of which confirm a lack of constriction in patients suffering from DM only compared to both other groups. Our finding is supported by a recently published study from Lott and colleagues, who have shown an impaired coronary and retinal arterial (and venous) vasoconstrictor response in patients suffering from DM compared to controls [31]. While this was a much smaller sample and applied a different methodology using a hyperoxic vs our flicker light stimulus, it confirms our results of an impaired ability of DM only patients to constrict to a physiologic stimulus. Our results of the SDRA and AUC analysis confirm a physiological difference in flicker light induced vessel dilation in DM. While the changes of the vascular system are present in all three groups, they can manifest with differing severity and hence partly explain this difference in behaviour.

Further explanations for the differences between groups that may be found in the underlying mechanisms involved in regulating vascular reactivity to flicker light include nitric oxide synthase and bioavailability [32], endothelial function [14] and ganglion cell signalling [33]. While NO synthase, NO bioavailability and endothelial health can be compromised in both DM and CVD, this is dependent upon disease duration as well as on medication. In this respect our finding of decreased arterial constriction following flicker light provocation in the DM only group is in agreement and expanding the data published by others [17, 34]. One group [33] found that this lack of arterial vasoconstrictor capacity was attributed to an alteration of nitric oxide production and bioavailability, whereas others [17] suggest that the overall reduced dilatory and constriction response could serve as an estimate of endothelial cell capacity to respond to a physiological stimulus. Furthermore, one study examining DM patients with varying severity of retinopathy demonstrated ganglion cell death with increasing severity of the retinopathy [35]. Our finding of more adverse endothelial markers in DM + CVD versus the other groups is in keeping with accepted pathophysiology [36, 37]. However, there were only three instances where levels of the markers correlated meaningfully with an ocular index, suggesting limited predictive value unless confirmed in a far larger population.

Some of the retinal parameters examined in the present study were comparable between the three groups. One explanation for this can be found in the fact that only six diabetic patients suffered from DR. Previous work examining retinal vessel calibres, carotid plaque and the impact of exercise on echocardiography parameters all confirmed that patients with DR compared to those suffering from DM without DR or CVD only had a higher risk of adverse CVD events and impaired myocardial function [38–40]. These findings are somewhat unsurprising given that with increasing duration of DM (and age) the prevalence of DR is increasing too, which is reflecting disease progression and a compromised microcirculation which in turn leads to a higher risk of adverse CVD events. As the majority of the patients assessed in the present study is free from DR this might explain in part why there were only subtle differences across groups. Observations over time taking into consideration DR onset will provide further insight into whether the differences in reaction pattern and the lack of arterial constriction to flicker light provocation in DM only patients is changing over time.

We acknowledge the artificial nature of the three groups, in that it is very likely that many of those we consider to have DM only will be carrying asymptomatic CVD. This also applies to the definition of DM, although we have strength in that our measurement of HbA_1c_ is robust and was performed on all subjects.

## Conclusion

In summary, patients with DM exhibit different retinal vessel reactivity patterns to flicker light provocation compared to those suffering from CVD only or a combination of both. While summarised (i.e. averaged across all flicker light cycles) retinal vessel diameters provide only limited capacity to distinguish between groups with different systemic vascular disease, dynamic vessel analysis using SDRA can do so if individual flicker light cycle patterns are analysed. Averaged retinal responses to flicker light lack the capacity to examine a change in reaction pattern and can mask changes in the reaction pattern. We note that only data with three complete cycle recordings should be used for average calculations as it would otherwise potentially distort the dilatory response especially in cases where there are differences between flicker cycles, such as demonstrated in our sample. Future research is needed to establish if retinal vessel reaction patterns change over time or with increased cardiovascular risk factors.

## Abbreviations

CVD: cardiovascular disease
DM: diabetes mellitus
NO: nitric oxide
IOP: intraocular pressure
DBP: diastolic blood pressure
SBP: systolic blood pressure
RVA: retinal vessel analyser
CRAE: central retinal artery equivalent
CRVE: central retinal vein equivalent
AVR: artery to vein ratio
BDF: baseline diameter fluctuation
MD: maximum dilation
MC: maximum constriction
DA: dilation amplitude
RT: reaction time
CT: constriction time
AUC_BASELINE_: area under the curve of the averaged 30 s prior to flicker start
AUC_20 s FL_: area under the curve of the averaged profile during 20 s flicker
AUC_20s Constriction_: area under the curve of 20 s duration 10 s following flicker cessation
AUC_Last 30s_: area under the curve of the last 30 s of the averaged reaction profile
SDRA: sequential diameter response analyses
